# Utility of regional STR marker variations in Tunisian and sub-Saharan populations: insights into forensic and population genetics

**DOI:** 10.3389/fbinf.2025.1550730

**Published:** 2025-06-17

**Authors:** Asma Attaoui, Hajer Foddha, Houcemeddine Othman, Hassen Ben Abdennebi, Amel Haj Khelil

**Affiliations:** ^1^ Laboratory of Human Genome and Multifactorial Diseases (LR12ES07), Faculty of Pharmacy, University of Monastir, Monastir, Tunisia; ^2^ Department of Genetics, Farhat Hached University Hospital, Sousse, Tunisia; ^3^ Laboratory of Cytogenetics, Molecular Genetics and Reproductive Biology (LR03SP02), Farhat Hached University Hospital, University of Sousse, Sousse, Tunisia; ^4^ Sydney Brenner Institute for Molecular Bioscience, Faculty of Health Sciences, University of the Witwatersrand, Johannesburg, South Africa; ^5^ Department of Molecular and Cellular Biology, Higher Institute of Biotechnology of Monastir, University of Monastir, Monastir, Tunisia

**Keywords:** allele frequency, genotype, power of discrimination, random forest, Y-STR

## Abstract

**Introduction:**

This study investigates the genetic variability and forensic applicability of Short Tandem Repeat (STR) loci including autosomal, X and Y-STR markers, across distinct Tunisian regions and among sub-Saharan African populations. Our objectives were to examine the regional allelic diversity of STR markers in Tunisia, and to assess the utility of these markers for forensic differentiation between Tunisian and sub-Saharan African.

**Methods:**

Twenty two STRs were genotyped in 500 Tunisian individuals and 501 sub-Saharan corpses by capillary electrophoresis using commercial system kits. A Chi-square test for homogeneity was applied to assess allele distribution and Principal Component Analysis to assess geographical allele variations. Bioinformatic methods in R packages were used, such as Logistic Regression Model to predict geographic group membership and Random Forest models to evaluate the discriminative power of the analyzed STRs.

**Results and Discussion:**

Statistical analyses revealed significant allelic variability between Northern, Central, and Southern Tunisia for markers such as D1S1656, D8S1179, and CSF1PO. PCA illustrated a clear genetic distinction between Tunisian and sub-Saharan populations, largely attributable to geographical and historical gene flow barriers. LRM achieved high accuracy (95.96%) in predicting geographic affiliation. RF analysis identified DYS391 as highly discriminative in population differentiation. Our findings align with prior research on Tunisian genetic diversity and extend this knowledge by illustrating allelic frequency variations in order to establish region-specific databases.

**Conclusion:**

This study contributes valuable insights into the genetic structure of Tunisian and sub-Saharan populations, emphasizing tailored approaches in forensic practices.

## Introduction

STR (Short Tandem Repeat) markers have become indispensable in human genetics due to their high variability, making them essential tools for individual identification in criminal cases and differentiation of populations and human migration. These markers are particularly valuable because they have a high degree of polymorphism, allowing geneticists to generate unique DNA profiles.

Fundamental works such as DNA Typing (Butler, 2005) showed how DNA can be used to identify suspects or victims with a very high degree of certainty and highlighted cases where DNA has helped solve complex investigations or exonerate people convicted of crime. The work of John Butler, has contributed significantly to the professionalization and reliability of forensic practices. By introducing standard techniques and rigorous protocols, he helped make DNA analysis a fundamental pillar in the judicial system, recognized for its high level of accuracy (Butler, 2023). Genotyping of autosomal STR has been, for a long time performed by capillary electrophoresis (CE) (Kaushik and Sahajpal, 2020). In addition to autosomal STRs those on the Y-chromosome are of special utility in criminal cases (Ruitberg et al., 2001; Ding et al., 2021; Barni et al., 2024).

Actually, numerous kits are developed to sequence a combination of autosomal-/X-/Y-STRs (Guo et al., 2017), identity single nucleotide polymorphisms (SNP) (Yagasaki et al., 2022) and mitochondrial genome (Holt et al., 2019), and can be expanded to include phenotype-and ancestry-informative SNPs. The use of massively parallel sequencing for forensic applications has expanded rapidly in the last few years allowing the transition from forensic genetics to forensic genomics supported by high throughput sequencing (Kayser and Parson, 2017; McCord and Lee, 2018; Haddrill, 2021; Kayser et al., 2023).

In addition to the great usefulness of STR markers in forensic investigations, genetic diversity studies deepened forensic genetics by providing unique information on regional genetic composition. The populations of North Africa, and particularly Tunisia, are of particular interest because of their rich historical mix of diverse populations, including Berber, Arab, Phoenician and sub-Saharan African groups. This mixture has resulted in distinct genetic profiles that distinguish North African populations from neighboring regions (El Moncer et al., 2010; Mejri et al., 2022).

Genetic diversity studies highlight the importance of creating region-specific genetic databases in forensic applications, as allele frequencies in North Africa differ significantly from those in Europe or the Middle East. Such databases are essential to improve the accuracy and reliability of forensic identifications in these populations (Frigi et al., 2014; Kostiuchenko et al., 1997).

In this study, we aim to, firstly, test on a large scale with regional comparison, the most discriminative markers in the Tunisian population, secondly, to assess the degrees of genetic relationships between Tunisian and sub-Saharan populations and, finally, to exploit results for application in the identification of Tunisian criminals and sub-Saharan migrant corpses.

## Materials and methods

### Sampling

This study involved two distinct samples collected in the Tunisian Scientific and Forensic Laboratories. The first sample included 500 individuals with criminal records, representing three geographic regions of Tunisia: North (133 individuals), Center (217 individuals), and South (105 individuals). Oral swabs (Milne et al., 2006) containing DNA from these individuals were gathered as evidence. The second sample consisted of 501 DNA samples extracted from the remains of sub-Saharan migrants recovered from Tunisian coastal areas. Both sample sets were pre-analyzed in line with established recommendations (Freeman et al., 2003). Samples were collected following requisitions by the relevant authorities (judicial, police). In the present study, ethical standards were meticulously followed throughout the sampling process. First, formal approval was obtained from our institution’s ethics committee prior to the commencement of any research activities. The confidentiality and anonymity of all data were vigilantly protected under the supervision of the Director of the Tunisian Forensic Science Department, who took proactive measures to mitigate any potential risks related to the unauthorized exploitation of participants’ personal information and biological samples. All sensitive data were handled with the utmost care and in strict accordance with prevailing legal and ethical regulations. Biological samples were collected in full compliance with established ethical principles and local laws, with particular emphasis on securing informed consent and protecting the rights of participants, especially those involved in legal proceedings.

### DNA extraction and genotyping

DNA extraction from oral swabs and genotyping were performed using the Investigator® 24plex QS Kit, Cat. No. 382415, QIAGEN, Hilden, Germany. This kit is designed for human identification, allowing directly multiplex amplification of 21 autosomal STR, one Y-STR and the Amelogenin marker for gender identification. It integrates an innovative quality sensor, which allows generating additional data that is very valuable for quality control and performance testing. In addition, it has high sensitivity and reliability with forensic samples (Hares, 2005).

DNA from human remains was extracted using the PrepFiler™ BTA Forensic DNA Extraction Kit, Applied Biosystems™, Catalog number 4463352, UK. This Kit has been developed for the extraction of DNA from calcified tissues (bone, tooth), as well as cigarette butts, tape lifts, and envelope flaps, thus increasing the potential to obtain probative information from downstream STR analysis. For genotyping, we used the PowerPlex® Fusion 6C System, PROMEGA, Madison, US, Catalog number DC2705. This system allows the amplification of 27 STRs including 25 autosomal loci, DYS391 and Amelogenin.

Allele values for each STR in the two samples were determined using the CE technology. Amplicons are separated by the 3,500 Genetic Analyzer, Applied Biosystems, UK, and analyzed using the Gene Mapper® ID version 3.2 software.

### Statistical and bioinformatic analyses

For the Tunisian sample, statistical analysis was performed using the Superior Performing Software System (IBM SPSS version 30.0.0.0 172) for MS-Windows. A Chi-square test for homogeneity was applied to assess allele distribution across Tunisia’s three regions (North, Center, and South). This test aimed to assess whether the allele distribution was homogeneous across the regions or whether there were differences between regions. Specifically, we compared the observed allele frequencies in each region, ensuring sample independence, with each individual being assigned to only one region. Before performing the test, we confirmed that the assumption of expected frequencies (greater than 5) was satisfied for each cell in the contingency table, thereby ensuring the validity of the results. The null hypothesis of this test posits that there is no significant difference in the allele frequency distributions across the three regions, while the alternative hypothesis is that there is a significant difference. A p-value of less than 0.05 was considered indicative of statistical significance.

For the expanded dataset (Tunisians and sub-Saharan individuals), we used the STRAF (STR Analysis for Forensics) R package (version 2.1.5) (Gouy and Zieger, 2017) with specific applications to autosomal STR data to calculate common forensic parameters. Forensic metrics were computed for the Tunisian and sub-Saharan African populations, as well as the combined cohort.

The data included a matrix of 22 STR markers. The Amelogenin was excluded as non-informative and missing values were addressed using the Simple-Imputer function from the scikit-learn Python library (version 1.5), with the imputation strategy set to the median number of repeats for each marker. To ensure that all features contributed equally to the subsequent analysis, the data were standardized to have a mean of 0 and a standard deviation of 1 using the ‘StandardScaler’ function. Principal Component Analysis (PCA) was then applied to reduce dimensionality while retaining maximum variance. Four principal components were computed, representing orthogonal directions of the highest variance in the data.

A logistic regression model was constructed to predict geographic group membership (Tunisian or sub-Saharan) based on the imputed matrix of the 22 STR markers. The model was trained using the ‘caret’ package (version 6.0.94) in R (version 4.3.3), specifying a binomial family with the generalized linear model method. This method models the probability that an individual belongs to one of the two geographical groups based on the allele frequencies of the STR markers. The dataset was split into training and test sets, with 80% of the data used for training and 20% for testing. Cross-validation was performed using 10-fold stratified cross-validation, ensuring that class probabilities were computed, and performance was evaluated based on the Area Under the Receiver Operating Characteristic curve (AUC). The ‘glm.control’ parameter was adjusted to allow for a maximum of 10,000 iterations to ensure model convergence. Model performance was assessed on the test set by generating predictions and class probabilities. A confusion matrix was used to evaluate classification accuracy, and the AUC was computed using the ‘pROC’ package (version 1.18.5) to quantify the model’s discriminative ability. The final AUC score was reported along with other performance metrics derived from the confusion matrix. A random forest model was additionally built to evaluate the discriminative power of the 22 STR markers, measured by the calculation of the decrease in Gini impurity, using the random forest package (version 4.7.1.1) in R. The number of trees was set to 500 (ntree = 500). For each split in the trees, 6 predictor variables were randomly selected and evaluated for the best split (mtry = 6).

## Results

All STR genotypes are provided in the Supplementary Sheet 2.

### Regional analysis in the Tunisian population

**FIGURE 1:**
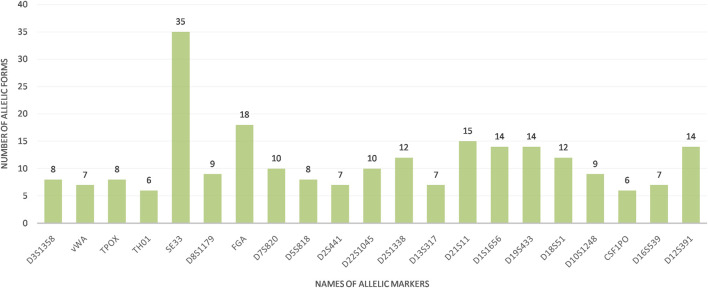
Allelic forms of the 21 STRs analyzed in the Tunisian population This figure is constructed using the Gene Mapper® ID version 3.2 software.

The calculation of allelic frequencies, based on relative fluorescence units (RFU) and statistical analysis, demonstrated variable allelic distributions and frequencies across the three Tunisian regions (North, Center, and South) when comparing the 22 STR markers (21 autosomal STR + Y-STR). This regional analysis revealed considerable allelic diversity, with a total of 236 distinct alleles identified and frequencies ranging from 0.005 to 0.051. Among these, 31 rare alleles (frequency <0.005) were observed, with the SE33 locus exhibiting particularly high polymorphism, featuring 35 alleles, 10 of which were classified as rare. In contrast, the THO1 and CSF1PO loci showed the least polymorphism, with only six alleles each (Figure 1; Table 1). The diversity results for the Y-STR marker DYS391 were not generated by the GeneMapper® ID version 3.2 software, unlike the other 21 bi-allelic markers, as the software assumes that a monoallelic marker has limited power to assess genetic diversity. In addition, our study elucidates unique regional allelic patterns, particularly at the D1S1656 locus in Southern Tunisia.

**TABLE 1:** Distribution of the 22 STR allelic forms in North, Center and South Tunisia. N: sample size.

Marker	Total N = 500		North N = 133		South N = 150		Center N = 217		*p* value
	Allele	Frequency	Allele	Frequency	Allele	Frequency	Allele	Frequency	
TH01	9 6 7	29.3 20.6 20.4	9 7 6	32.3 24.8 20.3	9 6 7	26.3 21 20	9 6 7	29.5 20.5 18.5	0.13
D3S1358	16 17 15	27.5 27 26.2	15 17 16	29.7 28.6 23.3	17 16 15	31 27.3 22	16 15 16	30.2 27 23.3	0.16
vWA	17 16 15	26.5 25 15	17 16 15	31.2 21.8 16.5	17 16 15	28.7 22.3 14.7	16 17 18	28.8 22.1 14.5	0.06
D21S11	29 30 32.2	23.8 23.8 12.5	30 29 32.2	23.7 19.9 13.9	29 30 32.2	26 23.3 13.7	29 30 28	24.7 24.2 14.7	0.48
TPOX	8 11 9	47.9 21.5 17.9	8 11 9	48.1 21.8 16.5	8 11 9	45.7 21.3 21	8 11 9	49.3 21.4 16.6	0.42
DYS391	9 11 10	40.4 27.2 26.6	9 11 10	50 27.7 18.5	10 9 11	45.8 32.6 20.1	9 11 10	39.7 31.9 18.1	**<0.001** ^a^
D1S1656	15 16 12 13	17.1 16.2 14.3 14.3	12 16 15	15.4 14.3 13.5	16 12 15	22 15.3 15	15 13 16	20.7 15.4 13.4	**<0.001** ^a^
D12S391	18 17 19	18.2 16.4 14.2	17 18 19	16.9 16.9 13.2	18 19 17	17.7 17.3 14.7	18 17 19	19.4 17.3 12.7	0.71
SE33	18 17 19	9 8.8 8.5	17 19 18	10.2 9 8.6	18 19 17	10.7 10.3 8	16 17 18	8.5 8.5 8.1	0.27
D10S1248	14 13 15	33.3 21.2 20	14 13 15	28.2 23.3 21.8	14 13 15	38.7 22 17.3	14 15 13	32.7 20.7 19.4	0.20
D22S1045	15 16 11	39.8 26.7 11.2	15 16 11	39.8 26.7 11.3	15 16 17	43.3 25 11	15 16 11	37.1 28.3 12	0.58
D19S433	14 13 15	24.8 23 13.7	13 14 15	24.4 23.7 14.7	14 13 15	26.7 26 14	14 13 12	24.2 20 13.1	0.10
D8S1179	13 14 15	22.4 18.9 17.1	15 13 11	23.3 20.3 15	13 15 12	25 19.3 13.3	13 15 14	21.9 18 17.3	**0.007** ^a^
D2S1338	17 20 19	31 17.2 12.3	17 20 19	29.5 16.3 13.1	17 20 19	34 20.4 8.7	17 20 19	29.4 15.3 13	0.47
D2S441	14 11 10	33.3 32.9 13.2	14 11 10	31.6 31.6 12	11 14 10	34.3 32.7 13.7	14 11 10	34.8 32.7 13.6	0.15
D18S51	14 16 13	14.3 14.2 14.1	17 12 14	15 14.3 13.9	14 16 12	18.7 17.3 13.3	13 15 12	16.6 14.3 14.3	0.25
FGA	23 22 21	15.1 12.4 12.4	23 22 21	15 13.9 12.4	21 23 22	12.4 13.7 11	23 22 21	16.1 12.4 9.7	0.10
D16S539	11 12 13	32.9 27 16.5	11 12 13	33.5 25.9 15.8	11 12 13	28.3 25 17.7	11 12 13	35.7 29 16.1	0.60
CSF1PO	10 12 11	31.7 30.7 28.7	10 11 12	36.1 30.1 24.1	12 10 11	35 29.7 25	12 10 11	31.8 30.4 30.4	**0.002** ^a^
D13S317	11 12 13	32.9 32.4 10.6	12 11 13	33.8 32 9	12 11 13	35.7 33.7 10.7	11 12 13	32.9 29.3 11.5	0.37
D5S818	12 11 13	36.2 21.8 18	12 11 13	30.5 27.1 18.4	12 13 11	30.5 27.1 18.4	12 11 13	38.7 21.4 17.3	0.11
D7S820	10 11 12	31 24.8 16.4	10 11 12	28.5 28.1 15.9	10 11 12	30.2 25.2 21.3	10 11 12	32.9 22.3 15.9	0.51

^a^ Significant difference between North, Center and South allele frequencies.

**FIGURE 2:**
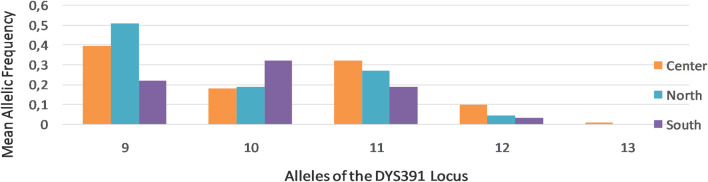
DYS391 allelic frequencies in the three regions of Tunisia: North (N = 133), Center (N = 217), and South (N = 105). 9–13 indicate the alleles of the DYS391 locus. This figure was constructed using the Gene Mapper® ID version 3.2 software.

Further analysis of STR markers revealed differential allelic frequency distribution across Tunisia’s regions for specific markers, such as DYS391 (Figure 2), underscoring the regional genetic variability.

The analysis highlighted high genetic diversity (GD), supported by elevated GD index values. Observed heterozygosity (Hobs) ranged from 0.625 to 0.938, while expected heterozygosity (Hexp) values ranged from 0.700 to 0.953 for TPOX and SE33 respectively (Table 2), indicating variations possibly due to inbreeding or migration within the population.

**TABLE 2:** Genetic diversity (GD) index of the 21 autosomal STR showing a gap between observed (Hobs) and expected (Hexp) heterozygosity.

Marker	GD index	
	Hobs	Hexp
CSFPO	0.677	0.720
D10S1248	0.792	0.794
D12S391	0.813	0.878
D13S317	0.646	0.780
D16S539	0.750	0.754
D18S51	0.865	0.877
D19S433	0.781	0.815
D1S1656	0.906	0.881
D21S11	0.750	0.831
D22S1045	0.656	0.752
D2S1338	0.813	0.851
D2S441	0.760	0.788
D3S1358	0.729	0.782
D5S818	0.667	0.760
D7S820	0.781	0.808
D8S1179	0.781	0.823
FGA	0.833	0.881
SE33	**0.938** ^a^	**0.953** ^b^
THO1	0.729	0.780
TPOX	**0.625** ^c^	**0.700** ^d^
VWA	0.729	0.818

^a,b^The highest GD index for Hobs and Hexp respectively; ^c,d^The lowest GD index for Hobs and Hexp respectively.

The polymorphism information content (PIC) values for each analyzed locus exceeded 0.6, with values ranging from 0.659 to 0.945 for TPOX and SE33 respectively, indicating the high polymorphism of these loci and their significant contribution to genetic variation in the Tunisian population.

The typical paternity index (TPI), which measures the STR marker’s ability to establish paternity, ranged from 1.333 to 8.000 for TPOX and SE33 respectively, demonstrating the markers’ strong utility in confirming biological relationships in paternity investigations within the Tunisian population.

The power of discrimination (PD), assessing a marker’s ability to distinguish between individuals, showed values ranging from 0.859 to 0.986 for TPOX and SE33 respectively, confirming that all STRs studied possess robust discriminative capabilities within the Tunisian population.

### Tunisian vs. sub-Saharan population analysis

**FIGURE 3:**
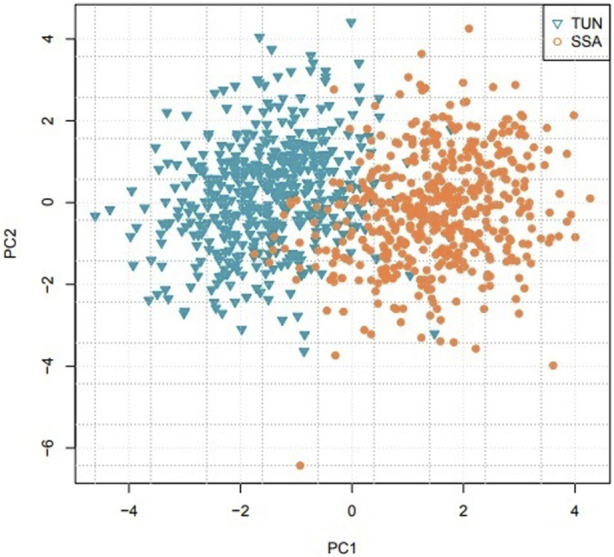
Principal Component Analysis comparing Tunisian (TUN) and sub-Saharan African (SSA) populations.

The relationships between individuals on the PCA projection tend to reflect their genetic relatedness. The closer individuals are on the PCA projection, the more genetically related they tend to be. In this work, PCA showed clear separation between Tunisian and sub-Saharan African populations when examining the first two principal components (PC1 vs. PC2), with a fixation index (FST) value of 0.0246, indicating significant genetic differentiation (Figure 3). However, differentiation diminished in higher-dimensional subspaces beyond the third principal component (Supplementary Sheet 1), suggesting that most of the variance between these populations was captured in the initial components. Some data points displayed overlap in the PC1 vs. PC2 plot, potentially due to individual variation, shared genetic traits, or errors in population group assignment during data collection.

The logistic regression model showed excellent predictive performance in classifying geographic group membership between Tunisian and sub-Saharan African populations. The confusion matrix revealed that the model correctly predicted 94 out of 96 sub-Saharan African (SSA) individuals and 96 out of 102 Tunisian (TUN) individuals. This resulted in an overall accuracy of 95.96% (95% CI: 92.19%–98.24%), significantly better than the No Information Rate (NIR) of 50.51%, with a P-value of <2e-16 indicating strong statistical significance. The Kappa statistic of 0.92 indicates a high level of agreement between the predicted and actual classifications. The McNemar’s test yielded a P-value of 0.3, suggesting no significant difference in the misclassification rates between the two classes. The recall of the model, which measures the model’s ability to correctly identify sub-Saharan African individuals, was 94%, while specificity, indicating the model’s accuracy in identifying Tunisian individuals, was 97.96%. The positive predictive value (PPV) was 97.92%, and the negative predictive value (NPV) was 94.12%. The prevalence of sub-Saharan African individuals in the dataset was 50.51%, with a detection rate of 47.47% and a detection prevalence of 48.48%. The balanced accuracy (F1 score), which accounts for both sensitivity and specificity, was calculated to be 95.98%. The model achieved an AUC score of 0.96, further validating its strong discriminative ability between the two geographic groups. These results indicate that the logistic regression model is highly effective in predicting geographic group membership based on the selected STR markers.

The random forest model which is used to measure the homogeneity of the groups formed during the data classification process, further identified the mean decrease in Gini values for different markers (Table 3). Notably, the DYS391 marker exhibited the highest values (140.85 and 149.00), underscoring its robustness in distinguishing between Tunisians and sub-Saharan Africans. This suggests that this marker facilitates the creation of more homogeneous genetic subgroups, thereby making distinctions between populations clearer and more reliable. This property is particularly advantageous in population genetics studies, where the ability to accurately distinguish subpopulations is crucial. Other markers, such as CSF1PO-A1 (8.94) and D18S51-A1 (12.86), also demonstrated relevance, although less than DYS391.

**TABLE 3:** Gini coefficient values of the 20 markers (19 autosomal and 1 Y-STR). D12S391 and D2S1338 are removed because of the lack of informativeness.

Marker	Mean decrease gini	
	A1 allele	A2 allele
CSF1PO	8.939	0.972
D10S1248	1.641	0.946
D13S317	3.070	1.905
D16S539	3.776	1.627
D18S51	12.859	4.455
D19S433	6.189	2.932
D1S1656	3.349	2.099
D21S11	1.327	2.377
D22S1045	1.209	2.233
D2S441	1.189	1.338
D3S1358	1.218	2.159
D5S818	1.150	1.080
D7S820	1.917	0.967
D8S1179	2.911	2.027
***DYS391***	***140.852*** ^a^	***149.002*** ^b^
FGA	2.290	3.773
SE33	2.103	8.359
TH01	2.575	4.172
TPOX	1.134	0.902
vWA	1.133	1.725

^a, b^Highest values of Gini coefficient for A1 and A2 alleles respectively.

## Discussion

In this study, we collected a sample size of 500 individuals. Although this sample size is sufficient for genetic studies, it represents only a small portion of the Tunisian population and does not capture its full genetic diversity, as it consists solely of individuals involved in legal cases. However, there are few, if any, studies indicating that STRs used in forensic science are influenced by socio-economic or criminal factors; rather, they are generally considered to be selectively neutral. As non-coding regions of DNA, they are not subject to the same selective pressures as coding genes. The variability of STRs is primarily due to random mutations, which makes their frequency independent of social or criminal behavior, and thus making them reliable for forensic analyses.

In the first part of this study, we expanded our analysis of autosomal loci from 21 (Bhambara et al., 2022) to 22 by incorporating the DYS391 as Y-STR. Due to its unique inheritance pattern, transmitted largely unchanged from father to son, mutations in DYS391 represent the primary source of its variation (Cockerton et al., 2012). This stability enhances its forensic applicability by enabling assessments of Y-STR evidence in criminal investigations using rapidly mutating Y-STR (Kayser, 2017), Y- single nucleotide variants (Zandstra et al., 2025) or combined with autosomal STRs using massively parallel sequencing applyed in Forensic DNA Phenotyping (Kayser et al., 2023). Alternatively, X-STRs uses are developed to address complex kinship cases (Gusmão et al., 2025).

The primary aim of our study was to conduct a comprehensive statistical analysis of the 22 STR genotypes. We identified the most discriminative allelic forms for each marker across Tunisian regions. Specifically, significant allelic distinctions were noted for markers DYS391, D1S1656, D8S1179, and CSF1PO, with *p*-values < 0.05. D1S1656 exhibited pronounced regional variation, especially between the North, South, and Center of Tunisia, with p < 0.001.

Our findings generally align with previous studies, though our population sample displays some distinct variations (Brinkmann, 1998; Ruitberg et al., 2001; Abdin et al., 2003; Butler, 2005; El Ossmani et al., 2007). According to Butler, the most polymorphic markers (FGA, D18S51, and D21S11) are distinguished by their complex repeat structures, which contribute to greater individual variability than simpler loci like TPOX and CSF1PO. In line with Butler’s observations (Butler, 2005), we also observed high heterozygosity in SE33 and D18S51, while D2S1338 displayed moderate discrimination between Tunisian regions in our dataset.

Ruitberg et al. identified SE33, FGA, D18S51, D21S11, and D2S1338 as highly discriminative markers based on mutation rates, whereas THO1 and TPOX ranked lower (Ruitberg et al., 2001). EL Ossmani et al. confirmed the significant discriminating power of 15 STR markers, with D18S51 being particularly prominent (El Ossmani et al., 2007). Similarly, our findings corroborate the discriminating capacity of D18S51, which revealed distinctive allelic frequencies across Tunisia’s North, South, and Center regions.

To enhance the generalizability of future studies and to gain a more nuanced understanding of the potential impact and limitations of selection bias, it is crucial to incorporate samples from diverse regions and a broader spectrum of social groups, particularly non-delinquent individuals, Nevertheless, when compared to other research on Tunisian samples from the general population (e.g., Cherni et al., 2005; Mahfoudh-Lahiani et al., 2006; Mejri et al., 2022), our findings reveal a striking similarity, thereby supporting the validity of our sample as a representative subset of the broader population.

Beyond forensic identification, we leveraged the 22 STR genotypes to examine the genetic divergence between Tunisian and sub-Saharan African populations. Logistic regression analysis demonstrated high efficacy in predicting geographic group affiliation based on these STR markers. PCA revealed a clear separation between Tunisian and sub-Saharan populations, reflecting significant genetic differentiation. This outcome aligns with established knowledge on African population genetics, where geographic and historical factors, such as the Sahara Desert, have acted as barriers limiting gene flow between North and sub-Saharan Africa. This inter-population variance was predominantly captured in the first two principal components (PC1 and PC2). However, higher dimensions (beyond PC3) revealed diminished discriminatory information, indicating that additional components contribute largely to minor intra-population variations.

In addition, the observed overlap between some data points on the PC1 vs. PC2 graph can be interpreted in several ways. One possible hypothesis is that these overlaps result from historical or contemporary genetic mixing between populations north and south of the Sahara. Indeed, several genetic studies have shown traces of gene flow between these regions, probably related to ancient migrations (especially *via* trans-Saharan trade routes) or to more recent population movements. Another explanation could be normal individual variation within populations, where some individuals share common genetic traits with those from other groups due to genetic heterogeneity specific to each population. Finally, it is also possible that these overlaps are partly due to errors in the classification of individuals during data collection. Further investigation of the selection criteria and sampling methods may help clarify these overlaps.

The logistic regression model demonstrated strong predictive capability, achieving an overall accuracy of 95.96%, significantly surpassing the NIR. With high sensitivity and specificity (94% for sub-Saharan identification and 97.96% for Tunisian identification), the model highlighted the discriminative strength of the selected markers for these populations.

The random forest model identified the most informative STR markers for geographic classification. DYS391 emerged as particularly influential, contributing significantly to the predictive power of the model. Markers such as CSF1PO-A1 and D18S51-A1, while still relevant, had lower importance scores, indicating they may capture more intra-population rather than inter-population variation. These findings demonstrate that effective population differentiation is contingent upon marker selection, with certain loci being inherently more informative in distinguishing groups.

This study offers valuable insights into the genetic differentiation between Tunisian and sub-Saharan populations, underscoring the utility of multivariate methods (PCA) and advanced classification techniques (LRM, RF). However, the observed overlaps indicate possible limitations, suggesting the existence of genetic complexities unaccounted for by our models, potentially attributable to historical gene flow or sampling biases.

Our results align with prior studies on Tunisian genetic diversity, which also highlight the substantial variation within this population. Indeed, during successive historical periods, Tunisia, by its strategic position in the extreme North of Africa has been a crossroads of multiple civilizations and their corresponding key population movements. Throughout its history, many people arrived and settled in Tunisia among the Berbers (Brett and Fentress, 1997). Tunisia, by its standing mid-way between the Eastern and Western Mediterranean, played a major role as a route for historical migrations. This resulted in the present Tunisian population being a mixture of multiple origins. Many studies used autosomal and X-STR loci to describe the Tunisian genetic heterogeneity (Frigi et al., 2014; Messoussi et al., 2019; Mejri et al., 2022). These results, added to the knowledge of the migration and occupation routes that occurred in the past all around the Mediterranean, give us a great opportunity to reconstruct the migration patterns. Indeed, previous studies provided genetic information relating to the mixed origin of the Tunisian population using Alu/STR markers (El Moncer et al., 2010), which revealed a sub-Saharan component probably due to sub-Saharan historical migrations as shown by previous analysis on the beta globin gene mutations (Bloom, 1995; Bennani et al., 1994).

A migration pattern similar to that of Tunisia has been observed in Libya, the Tunisia’s eastern neighboring, which was first inhabited, like Tunisia, by Berbers and then colonized by a variety of ethnic groups including Phoenicians, Greeks, Romans, Arabs and, more recently, Italians. A study, carried out on 175 Libyan males using haplotypes of 22 Y-chromosome-specific SNPs, revealed a predominant Northwest African component (signature of Berber speaking people, the autochthonous inhabitants) followed by one of a Middle Eastern origin (migration from Arabic populations). Overall, the comparative study with other populations (∼5,400 individuals from North Africa, Middle East, Sub-Saharan Africa, and Europe) revealed a general genetic homogeneity among North African populations (Triki-Fendri et al., 2015).

Other studies based on comparisons with sub-Saharan populations underscore Tunisians’ distinct genetic characteristics, with significant allelic differences at loci such as DYS391 and D18S51 (Caglià et al., 2003; Frigi et al., 2014). On a large scale, using sequence haplotypes and cutting edge statistical machinery, previous data on the Eurasian populations had shown that North and West African ancestry had entered Southern Europe, suggesting a key role for the Mediterranean in supporting gene flow back into Europe, consistent with migrations associated with the Arabic Conquest of the Iberian peninsula and earlier movements in and around Italy (Busby et al., 2015).

Finally, alongside the development of bioinformatic tools (Wang et al., 2022), we are advancing Next-Generation Sequencing (NGS) techniques as documented in recent literature (Guo et al., 2017; Butler, 2023). These technologies should permit expanding the sample size and incorporating additional markers, especially SNPs and mitochondrial. Employing advanced genomic technologies would further improve genetic resolution. Additionally, we aim to establish a region-specific STR database to enhance investigations of challenging cases, an approach already adopted in various populations (Ge et al., 2013; Caputo et al., 2023).

Currently, no public or centralized STR database exists for the Tunisian population. While several studies have provided valuable insights into the genetic diversity of Tunisia and neighboring countries, offering data on allele frequencies for STR markers, these data remain fragmented and are not compiled into a unified, accessible database. Our research is specifically designed to address this critical gap by compiling and analyzing an extensive STR dataset from the Tunisian population. Through the publication of these findings and, if possible, the integration of the data into a publicly accessible database, we aim to provide a reliable and invaluable resource for forensic practitioners. This effort will not only enhance the precision of forensic genetic profiling but will also significantly improve the interpretation of genetic data in forensic investigations, particularly within the Tunisian and wider Maghreb context.

Furthermore, integrating genetic, historical, demographic and anthropological data would provide a more complete picture of the genetic landscape, enhancing forensic capabilities and contributing valuable insights into human migration patterns across the Sahara and beyond.

## Conclusion

Overall, the combination of allelic frequency distribution, PCA, logistic regression, and random forest analyses provides a comprehensive view of the genetic diversity within the Tunisian population and its distinction from sub-Saharan African groups. The significant inter-population FST value, high discriminatory power of individual markers notably DYS391, and substantial TPI values indicate that the chosen STR markers effectively capture both intra- and inter-population genetic variation. This is particularly valuable for applications in forensic analysis and paternity testing within Tunisia and possibly across North Africa, where genetic markers can serve as reliable tools for identifying individual ancestry and familial relationships and predicting geographical origins.

The use of multiple statistical methods in this study not only reinforces the robustness of findings but also highlights the influence of specific loci on genetic diversity and population structure.

The study also highlights the need for comprehensive, population-specific databases to ensure accurate forensic identifications. We think that our study has direct forensic applications, particularly in the context of unidentified remains of clandestine migrants recovered along Tunisia’s coast. Many of these bodies, found in an advanced state of decomposition, make traditional identification methods impossible, as visual features are no longer distinguishable.

Our study offers a valuable tool in these situations. By establishing a genetic profile of the Tunisian population with 22 STR markers, we provide a means to identify the origin of remains, even when they are severely decomposed. This reference database could significantly aid forensic investigations, allowing for the differentiation between Tunisian and sub-Saharan populations, among others, in cases where visual identification is not possible. In light of the challenges posed by clandestine immigration, the development of such a database would enhance the accuracy of forensic identification, offering a more reliable method to identify victims and provide closure to families, while also assisting in legal and administrative processes.

Overall, a multidisciplinary approach integrating demographic, historical, anthropological, geographical and environmental data would provide deeper contextualization, enriching our understanding of human migration and genetic diversity.

## Data Availability

The original contributions presented in the study are included in the article/Supplementary Material, further inquiries can be directed to the corresponding author.

## Supplementary material

SUPPLEMENTARY FIGURE 1 Principal Component Analysis comparing Tunisian (TUN) and sub-Saharan African (SSA) populations beyond the third principal component: PC3 vs PC4. TUN: Tunisians; SSA: sub-Saharan Africans.

SUPPLEMENTARY TABLE 1 STR Genotypes of all samples imputed. TUN: Tunisians, NE: North-Est; NO: North-West; CE: Center-Est; CO: Center-West; SO: South-West; SSA: Subsaharan Africans, N: Number; Amel: Amelogenine marker.
