# Supplementary figures and images for: Utility of regional STR marker variations in Tunisian and sub-Saharan populations: insights into forensic and population genetics
Source: Front Bioinform. 2025 Jun 17;5:1550730. doi: 10.3389/fbinf.2025.1550730 (PMC12209214; doi:10.3389/fbinf.2025.1550730)

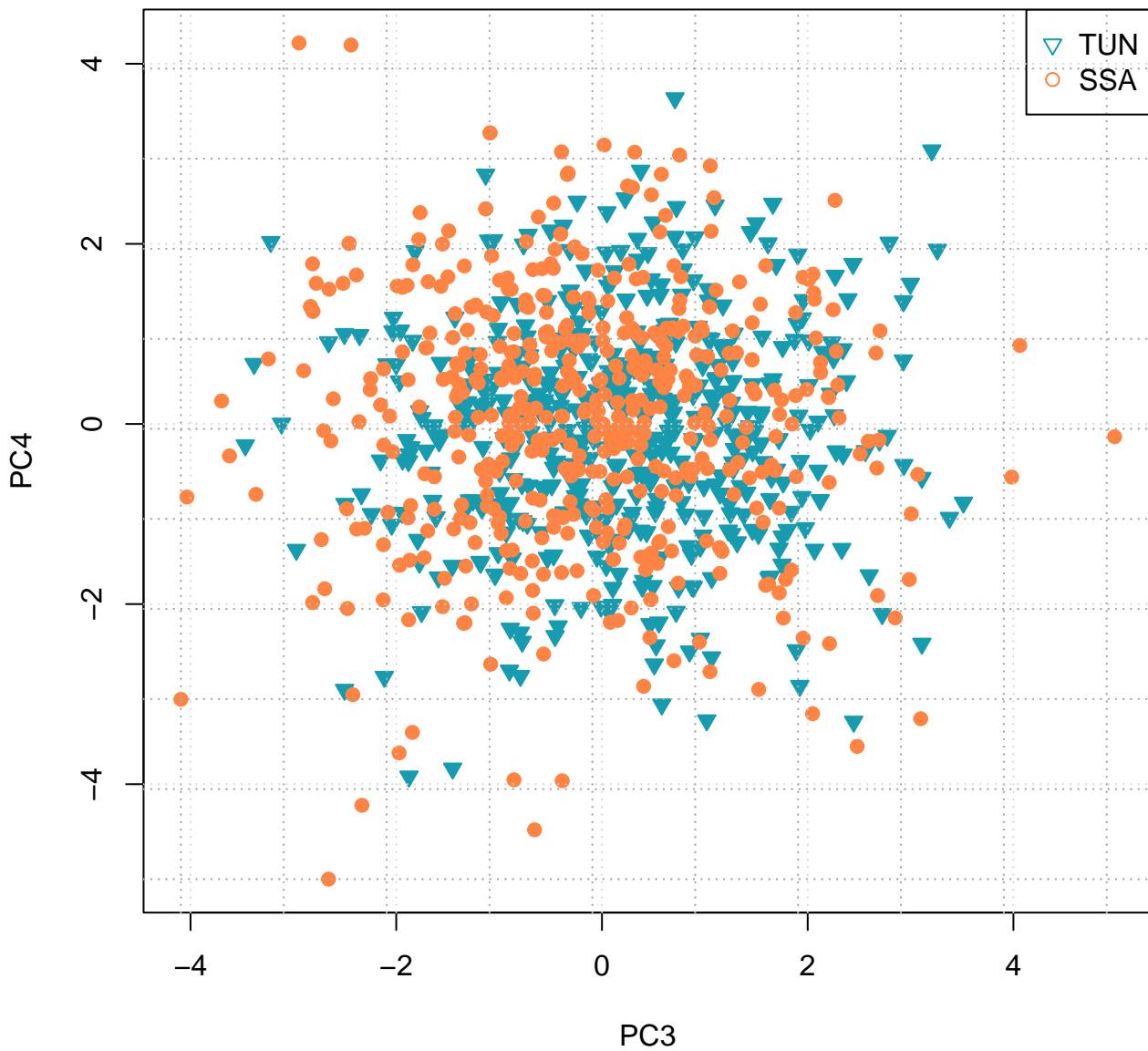

Supplement: Supplementary file 1 [file DataSheet1.pdf]
